# A kinetic model for RNA-interference of focal adhesions

**DOI:** 10.1186/1752-0509-7-2

**Published:** 2013-01-12

**Authors:** Max Hoffmann, Ulrich S Schwarz

**Affiliations:** 1 BioQuant, Heidelberg University, Im Neuenheimer Feld 267, 69120 Heidelberg, Germany; 2 Institute for Theoretical Physics, , Philosophenweg 19, 69120 Heidelberg, Germany

**Keywords:** Cell-matrix adhesion, Focal adhesions, RNA interference, Rac/Rho signaling pathways, Dynamic model, Parameter estimation, Timescales, Bifurcation analysis, Sensitivity analysis

## Abstract

**Background:**

Focal adhesions are integrin-based cell-matrix contacts that transduce and integrate mechanical and biochemical cues from the environment. They develop from smaller and more numerous focal complexes under the influence of mechanical force and are key elements for many physiological and disease-related processes, including wound healing and metastasis. More than 150 different proteins localize to focal adhesions and have been systematically classified in the adhesome project (http://www.adhesome.org). First RNAi-screens have been performed for focal adhesions and the effect of knockdown of many of these components on the number, size, shape and location of focal adhesions has been reported.

**Results:**

We have developed a kinetic model for RNA interference of focal adhesions which represents some of its main elements: a spatially layered structure, signaling through the small GTPases Rac and Rho, and maturation from focal complexes to focal adhesions under force. The response to force is described by two complementary scenarios corresponding to slip and catch bond behavior, respectively. Using estimated and literature values for the model parameters, three time scales of the dynamics of RNAi-influenced focal adhesions are identified: a sub-minute time scale for the assembly of focal complexes, a sub-hour time scale for the maturation to focal adhesions, and a time scale of days that controls the siRNA-mediated knockdown. Our model shows bistability between states dominated by focal complexes and focal adhesions, respectively. Catch bonding strongly extends the range of stability of the state dominated by focal adhesions. A sensitivity analysis predicts that knockdown of focal adhesion components is more efficient for focal adhesions with slip bonds or if the system is in a state dominated by focal complexes. Knockdown of Rho leads to an increase of focal complexes.

**Conclusions:**

The suggested model provides a kinetic description of the effect of RNA-interference of focal adhesions. Its predictions are in good agreement with known experimental results and can now guide the design of RNAi-experiments. In the future, it can be extended to include more components of the adhesome. It also could be extended by spatial aspects, for example by the differential activation of the Rac- and Rho-pathways in different parts of the cell.

## Background

Cells adhere to flat surfaces through focal adhesions, which are integrin-based contacts between the cell and the extracellular matrix [1-3]. Focal adhesions consist of more than 150 proteins with about 700 interactions [4,5]. Collectively they are known as the adhesome [6]. These proteins have been systematically classified in the adhesome project, which is accessible at www.adhesome.org. Many of the identified molecules are related to signaling [7], including signaling through the small GTPases Rac and Rho to the actin cytoskeleton. While Rho controls focal adhesion assembly and the concomitant formation of contractile stress fibers in the actin cytoskeleton [8], Rac was identified to be foremost responsible for the polymerization of an actin lamellipodium at a protruding cell edge and thus for the formation of nascent adhesions and focal complexes which typically assemble behind the protruding edge [9]. The main isoforms are RhoA and Rac1. GTPases are further regulated by guanine exchange factors (GEFs) and GTPase-activating proteins (GAPs). For the whole Rho-family, more than 130 different GEFs and GAPs have been reported [10]. The pathways of Rac and Rho have been modeled before in different contexts, for example circular dorsal ruffles [11], stress fiber contraction [12], membrane protrusion [13] or stress fiber alignment [14]. Rac is mainly acting through WAVE and Arp2/3 to activate polymerization of actin into dendritic networks required for protrusion. Rho promotes actin polymerization via the formin mDia1 and at the same time promotes myosin II contractility through ROCK and MLCP [15]. In general it is believed that Rac and Rho mutually inhibit each other [16-18], although recent data indicates a more complicated situation depending on the detailed temporal and spatial coordination of Rac and Rho within the cell [19].

**Figure 1:**
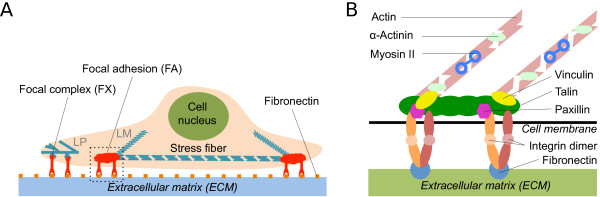
**Schematic presentation of focal adhesions.** Schematics of the situation of interest. **(A)** Cartoon of an adherent cell. During spreading and migration the cell adheres to ligands of the extracellular matrix (ECM), for example fibronectin, at the leading edge through nascent adhesions. They develop into focal complexes in the lamellipodium (LP), which can then mature into focal adhesions in the lamella (LM). Focal adhesions are typically connected to stress fibers that either run from one focal adhesion to another (ventral stress fibers) or end in the actin network (dorsal stress fibers). **(B)** Enlarged view of a focal adhesion with the main molecular components. The transmembrane protein integrin binds to the fibronectin on the ECM. The connection to the actin stress fibers, which can contract due to the myosin II motor molecules, is made by talin. This basic mechanical link is enhanced by proteins like vinculin, paxillin, or *α*-actinin.

Focal adhesions are not only signaling hubs, they also provide the mechanical linkage between the extracellular matrix and the actin cytoskeleton. For this purpose, they contain a large range of different connector proteins, including talin, vinculin, paxillin, and *α*-actinin. The spatial structure of focal adhesions has been extensively studied with fluorescence microscopy [20,21], revealing a layered structure dictated by the interplay of flat substrate and plasma membrane. The integrin layer is anchored in the extracellular matrix and therefore relatively immobile. The actin layer moves from the cell periphery towards the cell center driven by actin polymerization at the leading edge and myosin II contractility closer to the cell body. The connector layer moves backwards with the actin cytoskeleton, albeit with a reduced speed due to the effective friction with the underlying integrin layer. Although a more detailed picture of the spatial organization is still missing, recent advances with cryoEM [22], iPALM [23,24] and dual objective STORM [25] provide increasing insight. A schematic sketch of the situation of interest is given in Figure 1.

Focal adhesions are the result of a complex maturation process, which is strongly related to the overall spatial coordination in an adherent cell [26]. Nascent adhesions are thought to nucleate by integrin clustering underneath the lamellipodium, which is a relatively narrow region (1-3 *μ*m) at the cell periphery characterized by fast retrograde flow (≈25 nm/s) of rapidly polymerizing dendritic actin [27-29]. Towards the cell center, these nascent adhesions grow into focal complexes (FXs), which are sub-micron and typically round adhesions. Distal to the cell periphery, the lamellipodium gives way to the lamella, a relatively extended region characterized by more condensed actin structures, most prominently actin bundles contracted by myosin II motors [30]. Here the retrograde flow speed is reduced to ≈ 2 nm/s [28]. At the lamellipodium-lamella boundary, focal complexes either decay or become stabilized into mature focal adhesions (FAs) [31,32], which are micron-sized adhesion contacts typically elongated in the direction of the cell body.

The maturation of FXs into FAs has been shown to depend on the presence of physical force [33-37]. It is also strongly related to changes in molecular composition, in particular the recruitment of connector proteins such as vinculin and paxillin [38]. The correlation between force and maturation can be measured experimentally using traction force microscopy [34,39-41] and suggests that molecular checkpoints exist that ensure that FAs are only assembled if strong attachment is achieved. In 1978 Bell proposed that the rupture rate of a molecular bond under force is proportional to $e^{\left(F/F_{0}\right)}$, where *F* is the force acting on this bond and *F*_0_ an internal force scale of the order of pico-Newtons [42]. Thus, a higher force leads to a shorter lifetime. Bonds that follow this law have been termed *slip bonds*. It was believed that in general receptor-ligands pairs are slip bonds, although it has been pointed out that theoretically bond dissociation might also decrease under force [43]. During the last decade, several such *catch bonds* have been identified [44-46]. Most importantly in our context, the integrin-fibronectin bond has been shown to behave as catch bond [47]. This molecular feature might has evolved as part of the stabilization response of matrix adhesions under force. Because matrix adhesions are expected to consist of a mixture of different types of bonds, the two extreme scenarios would be pure slip bond versus pure catch bond behavior. Depending on their exact molecular composition, it is conceivable that adhesion clusters in different cell types, under different culture conditions and at different times of the maturation process behave more like slip bond or more like catch bond systems.

Focal adhesions are not only important for cell adhesion, but also for cell migration, division, and fate. Being essential for cell migration, they are key elements for many physiological and disease-related processes, including wound healing [48] and metastasis [49]. Recently they have been argued to be essential also for development [50] and stem cell differentiation [51]. There is a large range of possible mechanosensitive processes being involved at focal adhesions, including stress-sensitive ion channels, force-induced opening of cryptic binding sites and large-scale reorganization of the adhesions. For a systems level understanding of focal adhesions, it is mandatory to develop systematic procedures to assess the role of the different adhesome components.

One technique capable of such a systems level approach is the systematic use of RNA-interference (RNAi) [52-54]. In recent years RNAi-screens have become a standard tool in systems biology, as it allows us to dissect complex processes such as migration [55], division [56,57], or infection [58-60] in regard to the underlying molecular processes. The basic principle of RNAi is the following. Double stranded small interfering RNA (siRNA), which has a length of 21-23 nucleotides, is added to the cell using a variety of methods, for example by microinjection, electroporation or viral gene transfer [54]. During the following assembly of the RNA Induced Silencing Complex (RISC), the siRNA is separated into two strands, the guide strand and the passenger strand. The passenger strand is not needed any more and is degraded, whereas the guide strand is loaded onto the RISC complex. The siRNA-RISC complex then binds to the complementary target messenger RNA (mRNA). The bound target mRNA is degraded and released from the siRNA-RISC complex, which can then again bind other mRNA. The degraded mRNA can no longer be translated into proteins, and thus, the concentration of the protein is reduced in the cell. Until the maximum knockdown is achieved it typically takes 24-72 hours [61]. The stability of the knockdown depends mostly on the stability of the protein but also on factors like cell division rate or the degradation rate of the used siRNA. Therefore, the period of maximal knockdown can vary considerably.

First siRNA-screens have also been conducted for focal adhesions. In [62] numerous morphological features of cells and focal adhesions were analyzed and quantified for multiple siRNA. Here, it was suggested that several gene knockdowns caused similar effects and that many of the morphological features are strongly correlated. Recently a follow-up screen [63] highlighted the effect of specific knockdowns on the cell polarization response together with changes in focal adhesion formation and cell traction force. The authors suggest that both cell contractility and mechanosensing through focal adhesions are controlled by molecular checkpoints that regulate cell polarization.

In order to systematically and quantitatively understand the experimental results with their often counterintuitive relations, theoretical models for focal adhesions are required. In the literature several models for the force-mediated dynamics of focal adhesions have been proposed [32,64-69]. However, very few models make a connection to the molecular composition as revealed by the adhesome project. The compositional aspects of focal adhesions are represented best by kinetic models with a sufficiently large number of species. Such a model is the clutch model by Macdonald et al. [70]. In that model the focal adhesion is reduced to a three component model modeling the layered structure of the focal adhesions, representing integrin, actin, and a connector molecule that might be identified with e.g. talin. The temporal maturation of FXs into FAs is represented by modeling a hierarchical assembly in which these components successively assemble into a larger complex, which finally gets activated by force. For our purpose, such an approach is ideal to be expanded to include the effect of RNAi. However, because the clutch model focuses on the assembly aspect of focal adhesions, for a more comprehensive approach it has to be extended to include also the effect of signaling at focal adhesions.

Different models have been suggested to model the effect of RNAi. A very global view has been introduced by Bartlett & Davis, who published a model that consists of twelve ordinary differential equations [71]. They give a detailed description of not only the mRNA concentration and the protein concentration, but also take into account phenomena like extracellular transport, cellular uptake, cell division, and the subsequent reduction in siRNA concentration. Parameters were taken from the literature or were estimated. Their model has been verified by comparing the model’s results with a variety of in vitro data from the knockdown of luciferase. Apart from this global approach, several models have been suggested which focus on the core function of RNAi. Recently a systematic comparison of such model approaches has been conducted by Cuccato et al. [72]. They compared four of these coarse-grained models [73-75] with experiments conducted at human embryonic kidney cells. Fitting the models’ parameters to the experimental data suggested that the model originally proposed by Khanin & Vinciotti [74] fits best. This model is a purely phenomenological one that is based on a standard Hill-type kinetic model. A special feature of this model is that it saturates for high siRNA concentrations, which reflects the experimental findings by Cuccato et al. The most probable explanation for this effect is that the RISC-complexes (and/or other RNAi-associated complexes) are saturated with siRNA [76].

Here we introduce a kinetic model based on the clutch model by Macdonald et al. [70] which allows us to address many of the central questions related to RNAi of focal adhesions. We extend the original model to also describe translation and degradation of proteins, signaling to the actin cytoskeleton, and the detailed effect of force. Our paper is structured as follows. We first explain the model in the Methods section. In the Results and discussion section we discuss the dynamics of the system and the effect of RNAi on focal adhesions. With an analysis of the system dynamics we highlight the three different time scales present in the system. We then discuss more specific applications and extensions of our model. Finally a sensitivity analysis of the model enables us to pinpoint the effects of different knockdowns. We end with a short conclusion and with an outlook to possible future extensions of our model, especially in regard to spatial organization.

## Methods

### Model definition

**Figure 2:**
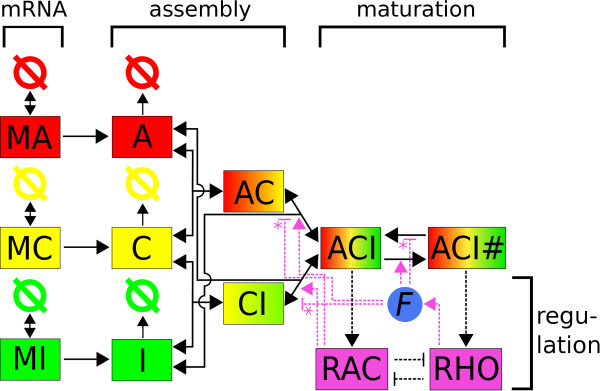
**Scheme of the kinetic model.** The overall model scheme reveals the main design principles. The mRNA transcription and degradation are depicted on the left hand side. After the translation of the proteins, these are assembled to focal complexes (ACI). Focal complexes can in turn mature to focal adhesions (ACI#). The assembly process is regulated by Rac and the maturation process is regulated by force and Rho. Depending on the force model used, the reactions marked with a star are either promoting (slip bonds) or suppressing (catch bonds) focal adhesion or focal complex disassembly.

In Figure 2 we schematically depict the suggested model. The three main design principles have been implemented. First focal adhesions are assembled in a hierarchical way. The proteins are translated from the mRNA and afterwards form intermediate complexes in a first assembly step before a second step leads to focal complexes. Second focal adhesions mature under force, which is accounted for by introducing a species for focal complexes (ACI) and one for mature focal adhesions (ACI#). Third the regulation of the assembly of both focal adhesions and focal complexes by the Rac/Rho pathways and their mutual inhibition is included.

Mathematically this scheme corresponds to twelve ordinary differential equations that will be explained in detail below. Due to the use of ordinary differential equations, we have no explicit spatial resolution, however, our choice of species and reactions includes an implicit spatial assembly order (layer-like structure) and mirrors the spatial segregation of focal complexes and focal adhesions. Modeling spatial processes with ordinary differential equations has been used successfully before, mainly in the context of compartment models. Examples in the context of cell adhesion are the original clutch model for focal adhesions [70] and a model focussing on the effect of Rac and Rho on focal complexes and focal adhesions [14].

Each differential equation in our model describes the dynamic behavior of one species. These species are: MA - messenger RNA for actin, MC - messenger RNA for the so called connector complex, which represents all proteins that are located between actin and integrin in the focal adhesion (most prominently talin), MI - messenger RNA for integrin, A - actin, C - connector complex, I - integrin, AC - complex where the connector complex is bound to actin, CI - complex where the connector complex is bound to integrin, ACI - full complex (focal complex), ACI# - mature full complex (focal adhesion), RAC - active Rac concentration, RHO - active Rho concentration. Apart from the mRNA part and the Rac/Rho regulation steps, we use mass action kinetics throughout our model.

#### RNA-interference

We start with a description of the messenger RNA (mRNA). The change in the concentrations of the mRNA is dependent on two processes: basal production rate with rate constant *k*_MX_ and basal degradation rate with rate constant *d*_MX_. An additional degradation term comes from the siRNA-treatment:

(1) $$\frac{\text{d}[\;\text{MA}]}{\text{d}t}=k_{\text{MA}}-d_{\text{MA}}[\;\text{MA}]-\delta ([\;\text{MA}],[\;\text{siRNA}])$$

(2) $$\frac{\text{d}[\;\text{MC}]}{\text{d}t}=k_{\;\text{MC}}-d_{\text{MC}}[\;\text{MC}]-\delta ([\;\text{MC}],[\;\text{siRNA}])$$

(3) $$\frac{\text{d}[\;\text{MI}]}{\text{d}t}=k_{\text{MI}}-d_{\text{MI}}[\;\text{MI}]-\delta ([\;\text{MI}],[\;\text{siRNA}])$$

The RNAi-term is implemented in our model in the following way:

(4) $$\delta ([\;\text{MX}],[\;\text{siRNA}])=d\frac{{\left[\;\text{siRNA}\right]}^{h}}{\Gamma^{h}+{\left[\;\text{siRNA}\right]}^{h}}[\;\text{MX}].$$

as suggested by Khanin & Vinciotti [74] and verified with human embryonic kidney cells by Cuccato et al. [72]. This ansatz is a standard Hill-kinetic model and is purely phenomenological. *d* is the maximum degradation rate of the mRNA due to RNA interference, while *Γ* is the concentration of siRNA needed to achieve half of the maximum degradation rate. *h* is the Hill coefficient.

In practice, usually only one component is knocked down. Then only the corresponding RNAi-term has to be included. The most reasonable choice seems to knock down the connector complex (for example talin), because integrins and actin are vital to the system. For example, cells lacking integrin are not capable to adhere on a 2D surface [77] and loss of *β*-actin is lethal for mice [78,79].

#### Assembly and maturation process

The next three equations describe the change in the concentrations of actin, connector complex, and integrin in their monomeric forms:

(5) $$\begin{array}{ccc} \frac{\text{d}[\;\text{A}]}{\text{d}t} & = & -\alpha_{\text{AC}}[\;\text{A}][\;\text{C}]+\delta_{\text{AC}}[\;\text{AC}]\\ & & -\alpha_{\text{A}}(1+\rho_{\text{RAC}}[\;\text{RAC}])[\;\text{A}][\;\text{CI}]\\ & & +\delta_{\text{A}}[\;\text{ACI}]+k_{\text{T,A}}[\;\text{MA}]-d_{\text{A}}[\;\text{A}], \end{array}$$

(6) $$\begin{array}{ccc} \frac{\text{d}[\;\text{C}]}{\text{d}t} & = & -\alpha_{\text{AC}}[\;\text{A}][\;\text{C}]+\delta_{\text{AC}}[\;\text{AC}]-\alpha_{\text{CI}}[\;\text{C}][\;\text{I}]\\ & & +\delta_{\text{CI}}[\;\text{CI}]+k_{\text{T,C}}[\;\text{MC}]-d_{\text{C}}[\;\text{C}], \end{array}$$

(7) $$\begin{array}{ccc} \frac{\text{d}[\;\text{I}]}{\text{d}t} & = & -\alpha_{\text{CI}}[\;\text{C}][\;\text{I}]+\delta_{\text{CI}}[\;\text{CI}]\\ & & -\alpha_{\text{I}}(1+\rho_{\text{RAC}}[\;\text{RAC}])[\;\text{AC}][\;\text{I}]\\ & & +\delta_{\text{I}}[\;\text{ACI}]+k_{\text{T,I}}[\;\text{MI}]-d_{\text{I}}[\;\text{I}]. \end{array}$$

Although in the cell actin can occur in many different forms, including monomeric, dendritic and bundled ones, for simplicity here we only introduced one species A; however, the different functions of actin are partially represented in the way this species interacts with the other ones. Likewise we introduce only one connector complex C, although in the adhesome, many different components might carry such a function. The concentration of the proteins A, C and I increases as they get translated from the accompanying mRNA or as higher order complexes disassemble. Degradation and incorporation into complexes decrease the concentration. Here and in the following equations, *δ*_X_ denotes the correspondent disassembly rate constant and *α*_X_ denotes the correspondent assembly rate constant. The translation and degradation rate constants of protein X are given by *k*_T, X_ and *d*_X_, respectively. The assembly of the intermediate complexes into focal complexes also depends on the Rac concentration, as Rac is known to promote the assembly of focal complexes [14]. We will focus on the dynamics of Rac and its counterpart Rho in the next section.

The rate equations for the intermediate complexes AC or CI are

(8) $$\begin{array}{ccc} \frac{\text{d}[\;\text{AC}]}{\text{d}t} & = & \alpha_{\text{AC}}[\;\text{A}][\;\text{C}]-\delta_{\text{AC}}[\;\text{AC}]\\ & & -\alpha_{\text{I}}(1+\rho_{\text{RAC}}[\;\text{RAC}])[\;\text{AC}][\;\text{I}]+\delta_{\text{I}}[\;\text{ACI}], \end{array}$$

(9) $$\begin{array}{ccc} \frac{\text{d}[\;\text{CI}]}{\text{d}t} & = & \alpha_{\text{CI}}[\;\text{C}][\;\text{I}]-\delta_{\text{CI}}[\;\text{CI}]\\ & & -\alpha_{\text{A}}(1+\rho_{\text{RAC}}[\;\text{RAC}])[\;\text{A}][\;\text{CI}]+\delta_{\text{A}}[\;\text{ACI}]. \end{array}$$

We note that these equations are generic for the first steps in the assembly of a three-component complex and that more specific assembly pathways can be implemented by a suitable choice of reaction constants. For example, if actin A cannot bind to the connector complex C because the complex CI has to form first in order to recruit actin polymerization factors like formin in order to assembly the ACI complex, one simply could set the reaction rate *α*_AC_ to zero. Here we refrain from such special choices, but in the results section we will comment on the effect of partially switching off one assembly pathway. For the fully assembled complex ACI we have

(10) $$\begin{array}{ccc} \frac{\text{d}[\;\text{ACI}]}{\text{d}t} & = & \alpha_{\text{A}}(1+\rho_{\text{RAC}}[\;\text{RAC}])[\;\text{A}][\;\text{CI}]-\delta_{\text{A}}[\;\text{ACI}]\\ & & +\alpha_{\text{I}}(1+\rho_{\text{RAC}}[\;\text{RAC}])[\;\text{AC}][\;\text{I}]-\delta_{\text{I}}[\;\text{ACI}]\\ & & -\tau_{\text{ACI}}[\;\text{ACI}]+\kappa_{\text{ACI}\#}[\;\text{ACI}\#] \end{array}$$

while for the force-activated mature complex ACI# we have

(11) $$\frac{\text{d}[\;\text{ACI}\#]}{\text{d}t}=\tau_{\text{ACI}}[\;\text{ACI}]-\kappa_{\text{ACI}\#}[\;\text{ACI}\#].$$

We note that apart from the equations (1)-(3) and the last two terms in equations (5)-(7), all terms appear at least twice, once with a positive sign and once with a negative sign. This is the direct consequence of our model mainly describing an assembly process. Thus, the only external fluxes in the system are the translation of new proteins from mRNA and the degradation of monomeric proteins. We also note that all but the translation reactions in the assembly system are reversible, as indicated in Figure 2. This represents the fact that all components are continuously reused in the cell. In the kinetic model, this important effect is represented by back reactions; for a more detailed model, one would have to introduce reservoirs channeling material from decaying focal adhesions back to growing focal complexes.

#### Rac and Rho signaling

As stated in the introduction, the Rac/Rho regulatory network is very complex [80] and as such, similar to the adhesome network, at the current state too complex to be modeled in full detail. Therefore, we only represent the main effects of the prominent isoforms Rac1 and RhoA. The activation of Rac and Rho depends in our model on the concentration of ACI and ACI#, respectively. Activation of Rac is assumed to be mainly correlated with the appearance of focal complexes, while Rho is activated in correlation with the appearance of focal adhesions [14,81,82]. Rac and Rho are coupled by a double negative feedback loop [15-18]. For our model we assume the inhibition of Rac by Rho and vice versa to be governed by a Hill function. We are only interested in the active forms of Rac and Rho and we assume the total amount of active and inactive Rac and Rho to be fixed. This leads to the following differential equations for the active Rac and Rho concentrations:

(12) $$\begin{array}{ccc} \frac{\text{d}[\;\text{RAC}]}{\text{d}t} & = & \kappa_{\text{RAC}}[\;\text{ACI}](1-[\;\text{RAC}])\\ & & -\frac{\beta_{\text{RAC}}[\;\text{RAC}]{\left(v[\;\text{RHO}]\right)}^{\gamma_{\text{RAC}}}}{K_{\text{RAC}}+{\left(v[\;\text{RHO}]\right)}^{\gamma_{\text{RAC}}}} \end{array}$$

(13) $$\begin{array}{ccc} \frac{\text{d}[\;\text{RHO}]}{\text{d}t} & = & \kappa_{\text{RHO}}[\;\text{ACI}\#](1-[\;\text{RHO}])\\ & & -\frac{\beta_{\text{RHO}}[\;\text{RHO}]{\left[\;\text{RAC}\right]}^{\gamma_{\text{RHO}}}}{K_{\text{RHO}}+{\left[\;\text{RAC}\right]}^{\gamma_{\text{RHO}}}} \end{array}$$

*κ*_RAC_ and *κ*_RHO_ are the parameters that determine how strongly the activation of Rac and Rho from its inactive forms depends on the concentration of the focal complexes and the focal adhesions, respectively. *β*_RAC_, *β*_RHO_, *K*_RAC_, and *K*_RHO_ are parameters that control the characteristics of the Hill function describing the double negative feedback. *γ*_RAC_ and *γ*_RHO_ are Hill coefficients and *v* is the feedback gain.

#### Effect of force

In the clutch model by Macdonald et al. [70], force is included as a dynamically changing parameter that is responsible for the transition from focal complexes to focal adhesions and the disassembly of both. Here we extend this approach by including the effect of the Rac and Rho signaling pathways. We introduce the force by the following changes in equations (8), (9), (10), and (11):

(14) $$\delta_{\text{A}}\to \delta_{\text{A}}\exp(F_{\text{r}}),$$

(15) $$\delta_{\text{I}}\to \delta_{\text{I}}\exp(F_{\text{r}}),$$

(16) $$\tau_{\text{ACI}}\to \tau_{\text{ACI}}F_{\text{r}}^{n}$$

(17) $$\kappa_{\text{ACI}\#}\to \varepsilon \frac{\delta_{\text{A}}+\delta_{\text{I}}}{2}\exp(F_{\text{r}})$$

with

(18) $$F_{\text{r}}=\frac{\chi +\rho_{\text{RHO}}[\;\text{RHO}]}{1+\sigma ([\;\text{ACI}]+m[\;\text{ACI}\#])}.$$

The first two substitutions describe the physical rupture of bonds under force. The exponential dependence on force [42] has been rationalized in terms of Kramers theory for escape over a sharp transition state barrier [83] and verified in single molecule experiments for a large range of slip bond systems [84]. Following [70], we assume that force increases the transition rate from focal complexes ACI to focal adhesions ACI# with a non-linear dependance. For our calculations we choose *n*=2. The disassembly rate constant of the focal adhesions is assumed to be the mean of the disassembly rates constants for the focal complexes multiplied by a factor *ε*, which describes how much more stable focal adhesions are compared to focal complexes.

The equation (18) for the force implements several important aspects of the system. *χ* can be interpreted as basal contractility level present even in the absence of activated Rho. Alternatively, in conjunction with equation (16), it can be interpreted as being related to the basal rate for the turnover of focal complexes to focal adhesions in the absence of force. The second term in the numerator represents the fact that force is mainly upregulated by active Rho. The denominator represents the fact that force is distributed and therefore diminished if adhesions are larger [42]. It has been shown experimentally that at focal adhesions, stress is higher by about a factor of three than at focal complexes (5.5±2 nN/*μ*m^2^ versus 2.0±1 nN/*μ*m^2^), possibly due to larger compactification of the focal adhesion [34,36]. We therefore weight focal adhesions with a factor of *m*=3. The factor *σ* describes the importance of the load sharing effect relative to a basal force level without load sharing.

Force has different effects on focal adhesions and our kinetic equations show that they tend to work in different directions: while focal complexes mature to focal adhesions under force due to a variety of molecular and physical effects, including changes in composition, the slip bond behavior tends to disrupt the higher order complexes on all levels. Catch bonds might have evolved in the context of cell adhesion to further counterbalance the disrupting effect of force. We explore this scenario in our model by changing the sign in the exponential functions in equations (14), (15), and (17):

(19) $$\delta_{\text{A}}\to \delta_{\text{A}}\exp(-F_{\text{r}}),$$

(20) $$\delta_{\text{I}}\to \delta_{\text{I}}\exp(-F_{\text{r}}),$$

(21) $$\kappa_{\text{ACI}\#}\to \varepsilon \frac{\delta_{\text{A}}+\delta_{\text{I}}}{2}\exp(-F_{\text{r}})$$

The functional form for the force and its effect on focal complex maturation remain unchanged in the catch bond model. We note that the pure slip and the pure catch bond cases are the two extreme cases which however are good indicators for the possible dynamics of focal adhesion assembly and knockdown. For this reason, we will mostly discuss these cases throughout the paper. Nevertheless we will comment on the effect of mixtures between slip and catch bonds later on in the results section.

### Initial conditions, parametrization and implementation

As will be reported in the results section, our model shows bistability between two states which are dominated by focal complexes and focal adhesions, respectively. Because these two states are stabilized by the Rac and Rho signaling parts, respectively, in the following we work with two sets of initial conditions (ICs) reflecting these two states. The **Rac-IC** has 1.0 for [ACI] and [RAC] while the **Rho-IC** has 1.0 for [ACI#] and [RHO]. If we include the RNA-part, [MA], [MC], and [MI] are initially set to 0.5. The initial concentrations of all other species will be set to zero.

For a complex kinetic model as presented here, it is difficult to completely explore parameter space and therefore the informed use of parameter values is crucial for model predictions. Macdonald et al. determined some of the parameter sets used in [70] based on a literature search. Afterwards these sets were sampled in steps of power of ten for each parameter to determine which of those parameter sets fit with experimental results [20,21]. In this way, a total of around 150 reasonable parameter sets was identified. Here we employ a slightly different strategy. First we have chosen three of these parameter sets by comparison with the experiments from [20], which suggested that in CHO cells the connector complex proteins are bound to actin about 30% of the measurement time. In our model, this corresponds to [ AC]/[ C_*total*_]= [ AC]/([ C]+[ AC]+[ CI]+[ ACI]+[ ACI*#*]) being around 0.3 [70]. Checking for results in this order of magnitude, we arrived at the three parameter sets given in the appendix. In order to explore a larger part of parameter space, we then interpolated 300 data sets with a power law:

(22) $$g(q)=g_{1}^{1-q}\cdot g_{2}^{q},$$

where *q* is a parameter that runs from 0 to 1, *g*_1_ is the value of a parameter from parameter set 1, and *g*_2_ is the value of the same parameter from set 2. *g*(*q*) is then the value of this parameter in an interpolated parameter set, determined by the value of *q*. Because the parameter sets from [70] did not result in the experimentally observed time scale for the dynamics of focal adhesion assembly (30-60 minutes according to [85]), we also modified the time scales of our reversible reactions (multiplication of both parameters with the same factor does not change the steady state).

All mRNA degradation rate constants were set to the same value for simplicity. Production and degradation rates were chosen as to achieve a typical concentration of 1. Protein translation rates were determined in a similar manner. Motivated by experimental observations, for the maximum degradation rate *d* we chose the value such that a final knockdown level of about 20% of the original mRNA concentration is achieved. A full siRNA mediated knockdown usually reaches its maximum efficiency after about 48 hours [61] and then keeps this low level for several days to weeks [71]. Thus, the time scale of the RNAi part of the model should be set to this time scale. Protein translation is a relatively fast process. In eukaryotic cells, translation of talin for example takes about 15-20 minutes at body temperature [86]. Thus, the knockdown time scale must be set by the additional degradation term caused by the siRNA presence. As explained above, there are many biological processes taking part in this knockdown which are not modeled in detail here, like siRNA-uptake or transport processes within the cell. We take care of this effect by introducing a hyperbolic time dependence for the siRNA-concentration:

(23) $$[\;\text{siRNA}](t)={\left[\;\text{siRNA}\right]}_{\text{MAX}}\cdot (1-\exp(-r_{\text{siRNA}}t)).$$

The parameter *r*_siRNA_ is chosen such that the steady state is reached approximately two days after beginning of siRNA-treatment.

The parameters for the Rho and Rac activation processes are chosen to be 1 for *β*_RAC_, *β*_RHO_, *K*_RAC_, and *K*_RHO_, a choice that has been made before for Rac/Rho systems [87,88]. The feedback gain *ν* is also set to 1. For the Hill coefficients we take *γ*_RAC_ = *γ*_RHO_ = 4 in order to achieve a sharp transition. In order to keep our model simple and reflecting the lack of information on the exact processes that lead to the activation of Rac and Rho at focal complexes and focal adhesions, we also set the remaining coupling parameters *κ*_RAC_ and *κ*_RAC_ to 1.

We used Mathematica (Wolfram Research Inc., Champaign, Il, USA, http://www.wolfram.com) to solve the system of twelve ordinary differential equations (ODE). The ODE system was numerically solved and the values for concentrations at *t* = 999999 were taken as steady state values. To ensure steady state, we compared with the values at *t* = 500000 and checked the summation theorem of the metabolic control analysis (see section Sensitivity analysis). All data plots were made with Mathematica.

## Results and discussion

### Model without RNA-part

**Figure 3:**
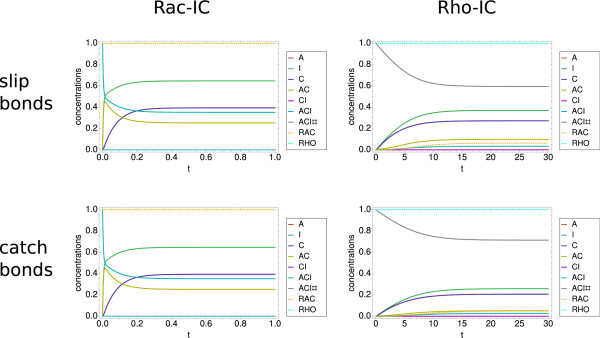
**Dynamics of the model without RNA-part.** Model without RNA-part. (Upper left panel) Slip bond model with Rac-IC. Note the very short time scale of below one minute for focal complex assembly, the high level of focal complexes and the very low (close to zero) level of focal adhesions. (Upper right panel) Effect of Rho-IC. Now the time scale is about 30 minutes. The result is a high steady state level of focal adhesions and a much lower level of focal complexes. (Lower left panel) Dynamics of the catch bond model with Rac-IC. The result is similar to the slip bond case due to the low level of force. (Lower right panel) Dynamics of the catch bond model with Rho-IC. The time scale remains the same as in the slip bond model, however, the steady state level of focal adhesions is noticeably higher as the focal adhesions are more stable in the catch bond case. Parameter set PS1 was used for these plots.

We first investigated our model without the RNA-part. By disregarding the external fluxes due to translation and degradation, we focus on the assembly part of our model. Then the three species A, C, and I have constant overall concentrations. In principle, the no-flux assumption reduces the number of independent variables and allows us to rewrite the system of equations. In order to allow comparison with the full model, however, here we keep the original definitions. We first investigated the slip bond model as shown in the two upper panels of Figure 3. In the upper left panel of Figure 3, we show the dynamic behavior of the slip bond system with the set of initial conditions corresponding to dominance of focal complexes (Rac-IC). With the parameter choice described above, the time axis units can be taken as approximately minutes. We show the time evolution in the first minute, as after this time period steady state is reached. This result is in line with studies of focal complexes that have identified a typical time scale of about one minute for focal complexes to assemble [89-91]. Although the concentration of focal complexes (ACI) initially decreases, it then plateaus at a relatively high level of ≈ 0.35. This is the result of Rac promoting focal complexes through a positive feedback loop. The high Rac concentration leads to very low Rho concentration due to the antagonistic behavior and in turn to a negligible amount of matured focal adhesions (ACI#). We conclude that the Rac-IC indeed lead to a state dominated by focal complexes.

In the upper right panel of Figure 3, we show the effect of using initial conditions corresponding to dominance of focal adhesions (Rho-IC). We first note the dramatic change in time scale: it now takes about 30 minutes for the steady state to be reached, in good agreement with experiments. A second important difference to the result with Rac-IC is that now the steady state concentration of focal adhesions is much higher (≈0.6). Rho suppresses Rac and, thus, as a consequence the amount of focal complexes is strongly reduced (≈0.03). However, in contrast to the first case, this level is not negligible. The reason for this is that the assembly of focal complexes is a necessary condition for their maturation into focal adhesions, thus, focal adhesions do not exist without focal complexes. We conclude that different system states are reached with different initial conditions, therefore, our model is bistable with the two possible states corresponding to low and high levels of focal adhesions stabilized by Rac and Rho, respectively. In the following, we will call these states Rac- and Rho-states, respectively.

We next investigated our model for the case of catch bond behavior as shown in the two lower panels of Figure 3. We first note that the lower left panel looks essentially unchanged to the upper left panel, indicating that the force model does not make a difference in the case of the Rac-state. The reason is that in this case the force is very low due to the low Rho-level, compare equation (18). In marked contrast, for the Rho-IC shown on the right side, the catch bond model makes a large difference. Now the steady state concentration of focal adhesions (ACI#) is about 20% higher compared to the slip bond case (≈0.71, while ACI slightly decreases to ≈0.02). This change is due to the catch bonds that increase the stability of focal adhesions under force. We conclude that the details of the force model are essential to predict the adhesion state of the system.

**Figure 4:**
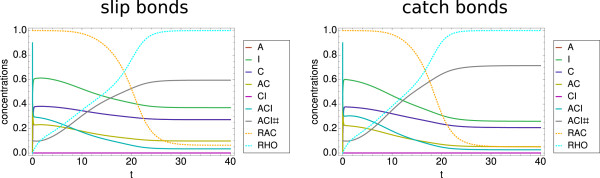
**Dependence on initial conditions.** Dynamics of the slip bond and catch bond model without the RNA-part and with initial conditions ACI=0.9, ACI#=0.1, RAC=1, RHO=0. Although there is only a small deviation from the Rac-IC, both models run into a steady state with a high amount of focal adhesions. Again the catch bond model leads to a higher amount of ACI# than the slip bond model. This sensitivity with respect to the initial conditions is very dependent on the parameter set. Both the sub-minute and the sub-hour timescales are visible in both plots. Parameter set PS1 was used for these plots.

We further investigated the robustness of our results in regard to variations of the initial conditions and found that the stability region for the Rac-state is relatively small. In Figure 4 we show the systems dynamics for initial conditions which have a somehow smaller initial level for focal complexes. First the focal complexes quickly adapt on a sub-minute time scale. Then the system develops into a focal adhesion state over the time scale of 30 minutes. This shows that the Rho-state has a relatively large region of stability compared to the Rac-state. This agrees with the above finding that a Rac-state does not have focal adhesions, but a Rho-state has focal complexes. While the first situation is reminiscent of fast cell migration, the second situation resembles slow migration or mature adhesion of strongly adherent cell types. We note that the detailed behavior depends on the choice of parameters.

**Figure 5:**
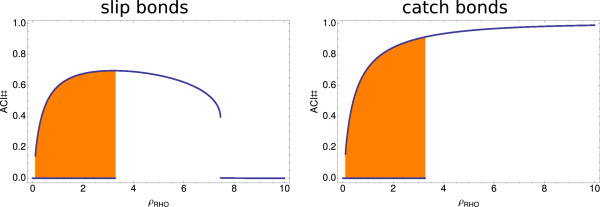
**Bifurcation analysis.** One-parameter bifurcation diagram showing the bistable region resulting from the two initial conditions (Rac-IC and Rho-IC) for the amount of focal adhesions (ACI#) in dependence of *ρ*_RHO_ that describes the influence of the Rho concentration on the force. Parameter set PS1 was used for these plots.

In order to further elucidate the bistable behavior of our system, we performed a bifurcation analysis. In Figure 5 a one-parameter bifurcation diagram for both the slip and catch bond models shows the stable steady state values for the amount of focal adhesions resulting from the two different initial conditions as a function of the value of the parameter *ρ*_RHO_, which describes the influence of the Rho-concentration on the force. For small *ρ*_RHO_, the system is bistable, leading to a high amount of ACI# for Rho-IC and a very small amount of ACI# for Rac-IC. Bistability is found for 0.15 < *ρ*_RHO_ < 3.3. For *ρ*_RHO_ > 3.3 the system is no longer bistable and both initial conditions lead to the same high amount of focal adhesions. The number of focal adhesions increases with an increasing value of *ρ*_RHO_ as subsequently the force and thus, ACI# increases. For large *ρ*_RHO_ the difference between the slip bond and the catch bond model becomes clear. In the slip bond model the system adapts a steady state characterized by a low amount of focal adhesions, as the high force levels disrupt the focal adhesions. Focal adhesions in the catch bond model however remain at a high amount as the increasing force decreases their disassembly rate. Thus, we conclude that our model is bistable between Rac- and Rho-states, and that catch bonds further stabilize the Rho-state.

### Full model with RNA and assembly parts

**Figure 6:**
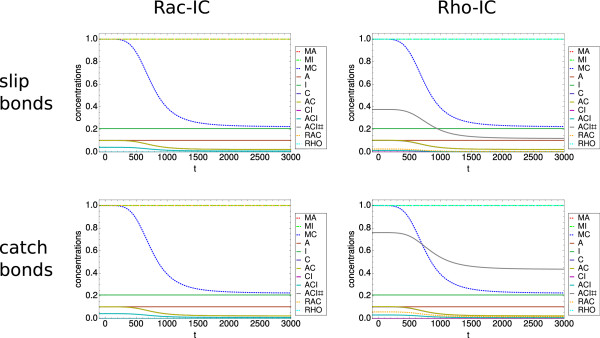
**Effect of RNAi.** Simulation of knockdown. (Upper left panel) Dynamics of the slip bond model with Rac-IC. The timescale of the siRNA mediated knockdown is about two days. Both the amount of focal complexes and focal adhesions is reduced by about 75-80%. (Upper right panel) Dynamics of the slip bond model with Rho-IC. The knockdown leads to a reduction of about 70% of the amount of focal adhesions. (Lower left panel) Dynamics of the catch bond model with Rac-IC. The result is comparable to the slip bond case. (Lower right panel) Dynamics of the coupled catch bond model with Rho-IC. Here the knockdown leads only to a reduction of about 40% of the amount of focal adhesions, which is considerably less than the 70% reduction in the slip bond case. Parameter set PS3 was used for these plots.

We now turn to the main focus of this paper, the effect of RNAi on focal adhesions. To this purpose, we now include the effect of RNA-synthesis, degradation and interference, that is we turn on the external fluxes. As motivated above, we focus on a knockdown of the connector molecules. We first note that due to the parameter choice of the translation and degradation reaction constants the steady states for the full model differ from the ones for the model without the RNA-part, thus, their exact values cannot be compared directly to the results from the preceding section. For the following analysis, we choose parameter values which allow us to explore all relevant regimes. In the upper left panel of Figure 6, we show a typical example for the time evolution of a system which has evolved from Rac-IC. Initially the system is in the steady state, although the ACI-level is now considerably lower than before. At *t*=0, the knockdown of the connector complex starts. In the first hours after the knockdown sets in, only a small effect can be seen. After approximately 500 minutes, a large effect sets in, which then levels out after approximately two days. The shape of the curve is mainly due to the Hill-type form of the delta term in equation (4). Note that the knockdown of MC happens on the time scale of about two days, however, the curves for C and all C-dependent complexes follow almost immediately, which is explained with the two shorter time scales of below one hour for both focal complex assembly and focal adhesion maturation that we investigated in detail for the no-flux model. The amount of focal complexes (ACI) gets reduced by about 75% and the amount of focal adhesions (ACI#) is lowered by about 80%, however, due to the already very low amount before the knockdown, the latter difference is not noticeable in the plot.

The same system with Rho-IC is shown in the upper right panel of Figure 6. The time scale of the knockdown remains the same, however, although the mRNA gets diminished by almost 80%, the amount of focal adhesions (ACI#) only gets reduced by about 70%, which again indicates that focal adhesions are more stable than focal complexes. Comparable to the no-flux model, the steady state level of focal adhesions remains much higher in this case (≈0.12).

In the lower left panel of Figure 6, we show a typical example for the time evolution of a system with catch bonds. Again the time scale remains the same. For Rac-IC the effect of the siRNA-mediated knockdown is almost undistinguishable from the slip bond model with Rac-IC. The reason is again the effect of low force that has been discussed in the section above. For Rho-IC we note that in the catch bond model the effect of the RNA interference is smaller. The level of fully assembled focal adhesions (ACI#) is higher than in the slip bond case before the knockdown due to the catch bonds increasing the stability of focal adhesions under force. Also the relative loss in the amount of focal adhesions is now only about 40%, which is considerably less than the 80% loss for the case with Rac-IC, but also less than the 70% loss for the slip bond model. These results support our conclusion that catch bonds behavior leads to a higher amount of stable focal adhesions.

**Figure 7:**
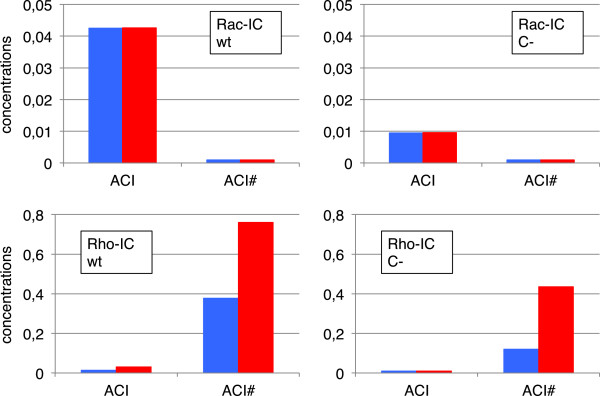
**Knockdown results summary.** Summary of steady state results for connector knockdown. The amount of ACI and ACI# is shown for wild type conditions and after the knockdown of the connector complex for both the slip bond (blue bars) and the catch bond model (red bars) with the two different initial conditions. In the upper panels, values below 0.001 are displayed as 0.001 and in the lower panels, values below 0.01 are displayed as 0.01. Focal adhesions are most stable under knockdown due to their stabilization through Rho-signaling. Stabilization is further increased by catch bond behavior. Parameter set PS3 was used for these plots.

In Figure 7 we summarize our results for the effect of knockdown. The left column shows the wild type results. For Rac-IC the system is in the Rac-state with a high amount of ACI and a low amount of ACI#. Due to the low resulting force level there is hardly no difference between the slip and catch bond models. For Rho-IC the system is in the Rho-state with a high amount of ACI# and a low amount of ACI. Now we see a large difference between slip and catch bond models, with the later one further stabilizing ACI#. A knockdown (right column) has clear effects. For Rac-IC, the reduction of the steady state levels of ACI# due to the knockdown is about 80%. For Rho-IC this reduction is smaller at about 70% for slip bonds and 40% for catch bonds. Thus, we conclude that a connector knockdown will have the largest effect on adhesion situations which involve focal complexes or focal adhesions dominated by slip bonds, whereas the effect will be rather mild for focal adhesions dominated by catch bonds. The prediction of a reduced amount of focal adhesions and focal complexes are in agreement with many of the knockdowns in the RNAi screens by Winograd-Katz et al. [62] and Prager-Khoutorsky et al. [63].

### Application to specific knockdowns

**Figure 8:**
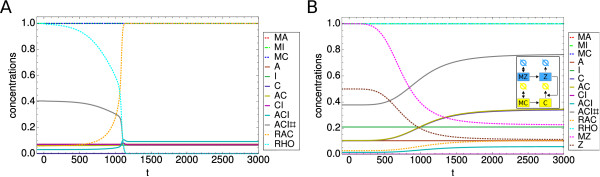
**Knockdown of Rho and of an additional regulating species.(A)** Effect of Rho knockdown. While the steady state amount of focal adhesions decreases, the amount of focal complexes increases due to the increased amount of Rac. The slip bond model with parameter set PS2 and Rho-IC was used for this plot. **(B)** Effect of a knockdown of an additional species Z acting on the degradation of C (see inset). Degradation of Z leads to an increased amount of C and a subsequent increase in the concentrations of both focal complexes and focal adhesions. The slip bond model with parameter set PS3 and Rho-IC was used for this plot.

Our model not only predicts situations in which the amount of adhesions goes down. One interesting example for the opposite effect is the knockdown of Rho or Rho signaling related components. We included this by reducing the total amount of Rho from 1 to 0.2 in a time-dependent manner representing the knockdown time scale. The results are shown in Figure 8A. It is immediately visible that while the knockdown of Rho leads to a reduction in focal adhesions (ACI#), Rac is no longer suppressed by Rho which in turn leads to an increase in focal complexes (ACI). This result is in line with the RNAi screens by Winograd-Katz [62] that found Rho and many Rho related knockdowns leading to a reduction in large adhesions but to an increase of small and round adhesions, which can be identified with the focal complexes in our model.

Another knockdown of interest requires a slight modification of the model which underlines the modular structure of our general model. We assume that the degradation of C is under the control of an additional species Z that itself is now knocked down. A scheme representing these changes can be found as an inset in Figure 8B. The ensuing knockdown dynamics is shown in Figure 8B. Knockdown of Z leads to an increased amount of C as its degradation is now reduced. This increase results in higher levels of both focal adhesions and focal complexes. One example for such a scenario might be proteolysis of talin by calpain 2 [92]. It has been shown by Bate et al. [93] that a mutation of talin that blocks cleavage by calpain 2 at a newly found binding site indeed leads to increased steady state levels of talin1. They also found larger adhesive area and a higher density of maturing adhesion as predicted by our model. Somehow different results were reported for a RNAi screens of focal adhesions [62], for which a knockdown of calpain 1, calpain 3, or calpain 7 leads to a decrease in large focal adhesions, whereas calpain 2 knockdown leads to an increase in large focal adhesions. In the future, our model can be used to further explore hypotheses in this context.

Another target of the additional regulator Z could be a member of the kindlin family of proteins. Kindlin is known as an integrin binding and activating protein [94] that is also able to bind to the actin cytoskeleton via migfilin [1]. Recent studies detailed the interaction between kindlin and integrin [95,96]. Zhao et al. [97] showed that calpain induced cleavage of kindlin 3 regulates cell adhesion and migration in hematopoietic cells. Cells with mutant kindlin 3 that is resistant to calpain cleavage showed higher adhesion levels. It was also shown that a calpain inhibitor (ALLM) leads to higher adhesion levels both in wildtype and mutant cells. Although these results are most probably cell-type dependent (as with the cleavage of talin), they show that calpain levels (which are represented by the additional species Z) play an important role in the regulation of focal adhesions through kindlins.

### More specific model assumptions

**Figure 9:**
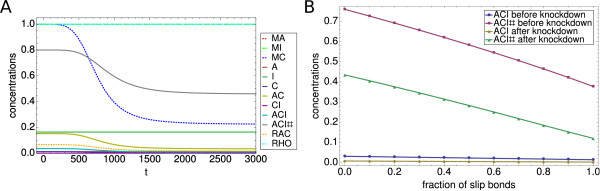
**Effect of a specific assembly pathway and of a mixture of slip and catch bonds.(A)** Effect of switching off the reaction of A and C to AC. The steady state values differ from before, however, the general picture remains the same, indicating that our model is capable of dealing with different pathway structures. The catch bond model with parameter set PS1 and Rho-IC was used for this plot. **(B)** Effect of a mixture of slip and catch bonds. The steady state amount of focal adhesion decreases almost linear with the fraction of slip bonds in the system. Results are shown for the full model before and after the knockdown. Parameter set PS3 and Rho-IC were used for this plot.

We next discuss the effect of making more specific assumptions on the assembly pathways. As mentioned in the methods section, assuming that CI needs to be assembled first to recruit actin polymerization promoting factors (e.g. formins and the Arp2/3 complex recruited to sites of adhesions) that allow actin to bind amounts to removing the reaction A+C from the system by setting the corresponding reaction rate to zero. The effect of this is shown in Figure 9A. We note that the dynamics differs from before, however, it does not change the general picture of the knockdown dynamics. This shows that our model presented here is a very general one and can be easily adapted to modified reaction schemes.

Another possible extension of the model is the consideration of mixtures of catch and slip bond behaviour. In Figure 9B we plot the steady state values for both focal complexes and focal adhesions before and after the knockdown as a function of the fraction of slip bonds in the system. Mathematically this is implemented as

(24) $$e^{F}\to e^{\text{sF}-(1-s)F}$$

in all appropriate differential equations, whereby *s* is the fraction of slip bonds. It is remarkable that the dependence of the steady state values on the fraction of slip bonds is almost linear. Thus, the relative reduction in focal adhesions due to the knockdown increases monotonically with an increasing number of slip bonds in the system, a tendency that was expectable from our results above for the pure slip and catch bond cases.

### Sensitivity analysis

In order to predict the effect of a knockdown in more detail, we next performed a sensitivity analysis based on ideas from metabolic control analysis [98-100]. In contrast to above, where we only discussed knockdown of the connector, for completeness we now address all three protein types (A, I, and C) and ask how the steady state value of focal adhesions depends on the degradation rates of the respective mRNAs. To this end we calculated concentration control coefficients (CCCs). The CCC of a steady state concentration $S_{i}^{\text{st}}$ of the *i*-th species with respect to the rate *ν*_*k*_ of the *k*-th reaction is defined in [100] as

(25) $$C_{k}^{i}=\frac{\nu_{k}}{S_{i}^{\text{st}}}\frac{\partial S_{i}^{\text{st}}/\partial p_{k}}{\partial \nu_{k}/\partial p_{k}}=\frac{\nu_{k}}{S_{i}^{\text{st}}}\frac{\partial S_{i}^{\text{st}}}{\partial \nu_{k}}.$$

Here the rate *ν*_*k*_ is defined as the difference in the forward and backward reaction rates and *p*_*k*_ can be any parameter that influences *ν*_*k*_. It is important for computations to choose the parameters in such a way that they have an influence only on one reaction, which means that *∂v*_*k*_/*∂p*_*k*_≠0 and *∂v*_*k*_/*∂p*_*l*_=0 for *l*≠*k*. A positive value of $C_{k}^{i}$ means that the steady state concentration of this species *i* increases if the rate *k* increases and a negative value indicates a decrease of the concentration of species *i* with an increased rate *k*. For the concentration control coefficients a summation theorem exists that states

(26) $$\overset{n}{\underset{k=1}{\sum }}C_{k}^{i}=0,$$

with *n* being the total number of reactions. We use this to verify our steady state values.

**Figure 10:**
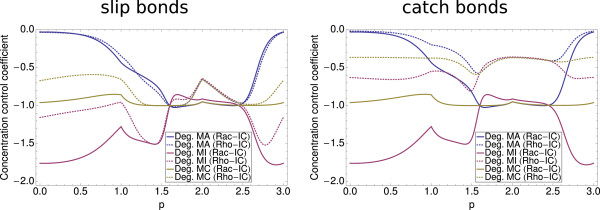
**Sensitivity Analysis.** Concentration control coefficients from sensitivity analysis. (Left panel) Concentration control coefficients (CCCs) for ACI# with respect to the degradation rates of the mRNA for the slip bond model for 300 different parameter sets. Both, for the Rac-IC as well as for the Rho-IC a knockdown of integrin would be most effective for the majority of the parameter sets. Nevertheless, there are parameter sets for which a knockdown of actin would yield the best results. The effectivity of the knockdown of the connector species remains constant for Rac-IC. For most parameter sets the focal adhesions are more stable for Rho-IC. (Right panel) Results for the catch bond model. For Rac-IC the result is comparable to the slip bond model. For Rho-IC, the absolute values of the CCCs are much smaller, indicating that the focal adhesions are much more stable if we assume catch bond behavior.

The results of the sensitivity analysis are shown in Figure 10, where we plot the CCCs for ACI# as a function of different parameter values. The 300 parameter sets contained in these plots were obtained by an interpolation between parameter sets PS1, PS2, and PS3 according to equation (22). The interpolation parameter p runs from 0.0 to 3.0 and the parameter sets from Appendix are located at *p*=0.0,1.0, and 2.0, respectively. The kinks at the integer values result from the fact that the direction of motion is changed in parameter space. Because in our model knockdown decreases the amount of focal adhesions, all CCCs presented here are negative.

In the left panel of Figure 10 we present the CCCs for ACI# with respect to the degradation rates of the messenger RNA for our slip bond model. For both initial conditions a knockdown of integrin would be most effective for the parameter sets with *p* < 1.6 and *p* > 2.4, as here the absolute value of the concentration control coefficient is the largest. This changes for 1.6 < *p* < 2.4, where a knockdown of the connector complex and actin would be more effective, however, only by a small margin for Rac-IC. The CCC for the knockdown of the connector complex remains relatively constant for all parameter sets. A plausible explanation is that it has always a similar effect because it is important for all possible (dis)assembly pathways with either AC or CI as intermediate complex. In general, for the two different initial conditions there are no qualitative differences, however, our analysis shows that in general the absolute values of the CCCs for all three species tend to be smaller for Rho-IC, indicating again that systems with a high number of focal adhesions are more robust towards siRNA-induced changes in protein concentrations.

The right panel of Figure 10 shows our results for the catch bond model. Again we find that the results are very similar for the Rac-IC. However, for Rho-IC the CCCs have much smaller absolute values due to the increased stability compared to the slip bond model. Not only are the absolute values the smallest, also the difference between the maximum and minimum values for each data set is the smallest. This confirms that focal adhesions with catch bonds are most robust, not only in regard to parameter variations, but also in regard to RNA-interference.

## Conclusions

In this paper we have presented a kinetic model to describe the effect of RNAi on focal adhesions. To this end we have combined model elements for siRNA mediated knockdown, focal adhesion assembly, force generation and regulation. We have successfully parametrized our model as to reproduce the three basic time scales relevant in this context, namely a sub-minute time scale for focal complex assembly, a sub-hour time scale for the adaptation of the focal adhesions to the changed environmental conditions, and a much longer time scale that is given by the time it takes the RNAi to be at its maximum level (roughly 48 hours).

Mechanical force plays different roles at focal adhesions. On the one hand, it physically disrupts focal adhesions, while on the other hand, it leads to a reinforcement effect. The second effect strongly depends on Rho-signaling. During recent years, it has been shown that stabilization of focal adhesions is also achieved by the peculiar property of some molecular bonds to become more stable under force (catch bonds). To explore the consequences of this feature, throughout the paper we have explored both a traditional slip bond model and a catch bond model. Our results strongly depend on these models, thus proving the importance of choosing the correct dissociation model under force.

Our first important result is that the system is bistable, with the two possible states differing in being either low or high in the amount of focal adhesions. Because these states are stabilized by the two positive feedback loops from the Rac- and Rho-pathways, we have called them Rac- and Rho-states. The typical initial conditions leading to these states are called Rac- and Rho-ICs. In general, the Rac-state is less stable and also more susceptible to RNA-interference. In contrast, the Rho-state is quite stable and also more robust in regard to RNA-interference, especially in the catch bond model. This main feature of our model is in line with the general view of the Rac-Rho-system leading to different and mutually exclusive cellular phenotypes [10].

A sensitivity analysis was used to systematically investigate the effect of RNAi over a large range of parameter sets. Independent of using Rac-IC versus Rho-IC or slip versus catch bond models, we found that a knockdown of integrins would be most efficient. However, because integrins are essential for proper cell function, it is more realistic to knock down the connector component, which we found to yield the second strongest effects on focal adhesions. We found that for Rho-IC in general the absolute concentration control coefficients were considerably smaller than for Rac-IC, especially in the catch bond case, in agreement with our earlier conclusion that the Rho-state is more stable than the Rac-state.

Our model now allows us to predict the effect of RNAi on focal adhesions, thus being a potentially very useful tool to guide corresponding experiments. For a given cellular system of interest, one first has to identify a parameter set for our model which best corresponds to the experimental system. Using explicit integration or the sensitivity analysis, one then could predict the most efficient strategy to knockdown specific features of the system, for example focal complexes or focal adhesions. Using the examples of Rho and calpain with its effects on talin and kindlin, we have shown how our model can be adjusted to more specific situations of interest.

One important aspect emerging from our model is the role of initial conditions. Bistability leads to the effect that the choice of initial conditions becomes important. Throughout this work we have therefore distinguished between Rac- and Rho-ICs. A practical consequence of this finding is that in experiments one has to differ between setups in which knockdown has been performed after or before the last plating step. Because spreading (like migration) corresponds to the Rac-state, while mature adhesion corresponds to the Rho-state, a knockdown during mature adhesion might have much less effect than a knockdown before replating after trypsination. In the future our model can be used to pursue this aspect further and to investigate whether there is a difference in the results between the two fundamentally different ways to implement a knockdown.

There are several limitations to our model which might be addressed in future work. In order to establish the appropriate conceptual basis for our system of interest, we have focused on three generic protein components, thus neglecting further known details of the complex composition of focal adhesions. In the future, the model could be complemented by more detailed models for the hierarchical structure of adhesion contacts, for example the interplay between integrins, talin, vinculin and the actin cytoskeleton. For actin, a more detailed modelling might introduce different species to account for its different functional contexts (monomeric in the cytoplasm, dendritic in the lamellipodium and bundled in the lamella). Then the model might also be extended by a more explicit model for actin polymerization, including species representing formins or Arp2/3.

Another major limitation is the restriction to a kinetic approach, assuming a well-mixed system. Although our model represents the layered nature of adhesions and the segregation into focal complexes and focal adhesions, it does not represent their complex spatial coordination. Our approach does not account for the number, spatial distribution, size or shape of the adhesion sites, but only makes statements on the average phenotype expected for different conditions, including knockdowns. Future work is required to include the spatial dimension, either by using partial differential equations or particle-based simulations. In the future, our approach might be combined with detailed spatial models for the localization, size, and shape of adhesion sites [101,102].

To conclude, our work introduces a flexible modeling framework for cell-matrix adhesions which represents many of their biochemical and physical features as they are currently known. It is especially suited to study the effect of RNA-interference and makes specific predictions about its effectiveness. Thus, it is an ideal starting point to guide and analyze corresponding experiments.

## Appendix

The basic parameter sets (PS) used in the model are listed in Table 1.

**Table 1:** Basic parameter sets (PS) used in the model

**Focal adhesion parameters**				
**Parameter**	**PS1**	**PS2**	**PS3**	**Source**
*α*_A_	10000	1000	10	[70], this paper
*δ*_A_	1	100	1	[70], this paper
*α*_I_	100	1000	100	[70], this paper
*δ*_I_	100	100	100	[70], this paper
*α*_AC_	0.1	0.01	100	[70], this paper
*δ*_AC_	10	10	10	[70], this paper
*α*_CI_	10	10000	0.01	[70], this paper
*δ*_CI_	100	10	100	[70], this paper
*τ*_ACI_	10	10	10	this paper
*ε*	0.001	0.001	0.001	this paper
*χ*	0.001	0.001	0.001	this paper
*σ*	1.0	1.0	1.0	this paper
**RNAi parameters**				
**Parameter**	**PS1**	**PS2**	**PS3**	**Source**
*k*_MA_	17.3	17.3	17.3	this paper
*k*_MC_	17.3	17.3	17.3	this paper
*k*_MI_	17.3	17.3	17.3	this paper
*d*_MA_	17.3	17.3	17.3	this paper
*d*_MC_	17.3	17.3	17.3	this paper
*d*_MI_	17.3	17.3	17.3	this paper
*k*_T,A_	2.07681	0.735221	1.03706	this paper
*k*_T,C_	2.07437	0.0206286	1.03476	this paper
*k*_T,I_	2.59193	0.652918	2.08517	this paper
*d*_A_	10	10	10	this paper
*d*_C_	10	10	10	this paper
*d*_I_	10	10	10	this paper
*d*	60.0	60.0	60.0	this paper
[ siRNA]_MAX_	1.0	1.0	1.0	this paper
*r*_siRNA_	10^−4^	10^−4^	10^−4^	this paper
*Γ*	0.1	0.1	0.1	this paper
*h*	4.5	4.5	4.5	[72]
**Rac & Rho parameters**				
**Parameter**	**PS1**	**PS2**	**PS3**	**Source**
*κ*_RAC_	1.0	1.0	1.0	this paper
*κ*_RHO_	1.0	1.0	1.0	this paper
*β*_RAC_	1.0	1.0	1.0	[87,88]
*β*_RHO_	1.0	1.0	1.0	[87,88]
*K*_RAC_	1.0	1.0	1.0	[87,88]
*K*_RHO_	1.0	1.0	1.0	[87,88]
*γ*_RAC_	4	4	4	this paper
*γ*_RHO_	4	4	4	this paper
*v*	1.0	1.0	1.0	this paper
*ρ*_RAC_	1.0	1.0	1.0	this paper
*ρ*_RHO_	1.0	1.0	1.0	this paper

## Abbreviations

FX: Focal complex; FA: Focal adhesion; RNAi: RNA interference; A: Actin; C: Connector complex; I: Integrin; AC: Actin-connector complex; CI: Connector-integrin complex; ACI: Full complex; ACI#: Mature full complex; Rac-IC: Rac initial conditions (ACI(0)=Rac(0)=1, ACI#(0)=Rho(0)=0); Rho-IC: Rho initial conditions (ACI(0)=Rac(0)=0, ACI#(0)=Rho(0)=1); Z: Additional regulatory species

## Competing interests

The authors declare that they have no competing interests.

## Authors’ contributions

MH and USS developed the model and MH conducted the numerical analysis. MH and USS wrote the manuscript. Both authors have read and approved the final manuscript.
